# Quality of occlusal outcome in adult class II patients after maxillary total arch distalization with interradicular mini-screws

**DOI:** 10.1186/s13005-024-00425-1

**Published:** 2024-04-26

**Authors:** Yann Janssens, Patrick F. Foley, Frauke Beyling, Rainer Schwestka-Polly, Jonas Q. Schmid

**Affiliations:** 1 Department of Orthodontics, Université Paris Cité, Paris, France; 2 Department of Orthodontics, Saint Louis University, St Louis, MO USA; 3 Private Practice, Bad Essen, Germany; 4 Department of Orthodontics, Hannover Medical School, Hannover, Germany; 5 Department of Orthodontics, University of Münster, Münster, Germany

**Keywords:** mini-screws, Interradicular mini-screws, Maxillary total arch distalization, ABO score, Class II correction, Lingual orthodontics, Completely customized lingual appliance

## Abstract

**Background:**

The aim of the investigation was to evaluate if a Class II malocclusion in adult patients can be successfully corrected by maxillary total arch distalization with interradicular mini-screws in combination with completely customized lingual appliances (CCLA).

**Methods:**

Two patient groups were matched for age and gender to determine differences in the quality of final treatment outcome. The treatment results of 40 adult patients with a Class I malocclusion (Group 1) were compared with those of 40 adult patients with a moderate to severe Class II malocclusion (Group 2). All patients had completed treatment with a CCLA (WIN, DW Lingual Systems, Bad Essen, Germany) without overcorrection in the individual treatment plan defined by a target set-up. To compare the treatment results of the two groups, 7 measurements using the American Board of Orthodontics Model Grading System (ABO MGS) and linear measurements for anterior-posterior (AP) and vertical dimensions were assessed at the start of lingual treatment (T1), after debonding (T2B), and compared to the individual target set-up (T2A).

**Results:**

A statistically significant AP correction (mean 4.5 mm, min/max 2.1/8.6, SD 1.09) was achieved in Group 2, representing 99% of the planned amount. The planned overbite correction was fully achieved in both the Class I and Class II groups. There was a statistically significant improvement in the ABO scores in both groups (Group 1: 39.4 to 17.7, Group 2: 55.8 to 17.1), with no significant difference between the two groups at T2B. 95% of the adult patients in Group 1 and 95% in Group 2 would meet the ABO standards after maxillary total arch distalization with a CCLA and interradicular mini-screws.

**Conclusions:**

CCLAs in combination with interradicular mini-screws for maxillary total arch distalization can successfully correct moderate to severe Class II malocclusions in adult patients. The quality of the final occlusal outcome is high and the amount of the sagittal correction can be predicted by the individual target set-up.

## Introduction

Class II malocclusion correction in adult patients can be performed in many ways. Comprehensive diagnostics are mandatory if a non-surgical approach is chosen, as dentoalveolar correction can be performed primarily in the maxilla by premolar extractions or distalization, or at the mandible by intermaxillary Class II elastics or by rigid/flexible fixed functional appliances. When selecting the most suitable approach, the reliability and efficiency of the various treatment concepts are among the main considerations. Today, mini-screws (MSs) are considered a reliable anchorage system with a wide range of indications [1]. They allow non-interradicular insertion away from the teeth, such as in the palate, and interradicular placement [2–7]. When an insertion in the anterior palate for maxillary total arch distalization (MTAD) is selected, a supra-construction will be required in most cases, which is now routinely digitally designed and fabricated using an additive manufacturing process (selective laser melting) [8]. A less complex concept is MTAD using interradicular MSs combined with fixed labial appliances, as first introduced by South Korean authors [6, 9]. In a recent study, Beyling et al. have shown preliminary results using this concept in combination with completely customized lingual appliances (CCLAs) mostly in adolescent patients [10]. It was found that reliable levelling of the mandibular curve of Spee, along with controlled mandibular incisor intrusion and reliable control of maxillary incisor root torque before and during the retraction are essential for a successful Class II correction.

Recently, some authors have investigated the possibility of Class II correction in adult patients using clear aligners in combination with intermaxillary Class II elastics in a retrospective analysis, comparing the treatment results with a control group of age- and gender-matched patients with Class I malocclusions [11, 12]. The American Board of Orthodontics Model Grading System (ABO MGS) was selected as the outcome measure and the focus was on the quality of the occlusal treatment outcome. The method introduced by Patterson et al. was also used in this study because it clearly illustrates the quality of the final treatment outcome that can be achieved with the chosen approach [11].

Testing was performed against the null hypothesis that there is a significant difference in the quality of treatment outcome, as defined by the ABO MGS score, between a group of patients with a moderate to severe Class II malocclusion treated with CCLAs and MSs for MTAD and a Class I group also treated with CCLAs.

## Material and methods

The approval for this retrospective cohort study was received from the ethical committee of the Hannover Medical School, Hannover, Germany (3151–2016). Inclusion criteria were adult patients 18 years of age or older at the onset of lingual treatment with Class I or II malocclusion who were consecutively treated with a CCLA (WIN, DW Lingual Systems, Bad Essen, Germany) in one orthodontic specialist practice (Bad Essen, Germany), and were debonded between 2019 and 2023. Patients with a known centric occlusion-centric relation discrepancy, planned extractions and space closure, dental bridges, or a compromised treatment plan where the target set-up did not represent a Class I were excluded. History of previous orthodontic treatment, missing teeth, missing records or bad compliance (e.g.: bad oral hygiene, compromised Class II elastic wear during night time, missing appointments) were not exclusion criteria. All fixed lingual treatments were completed by orthodontic specialists with high expertise in the field of CCLA treatment.

Two groups were defined: Group 1 with Class I malocclusion, and Group 2 with Class II malocclusion. Inclusion criteria for the Class II group was at least half-a-unit Class II occlusal relationship on one side. Groups were classified using ABO classifications for molar relationship. Even distribution in both groups was ensured by matching age and gender of patients with Class I to the included patients with Class II. An ideal occlusion without overcorrection was defined for all included cases as the goal for the target set-up process, as it is known that fixed orthodontic appliances can deliver full three-dimensional control [13–15]. The individual set-up was made on plaster models ensuring a really three-dimensional view for the dental technicians.

**Fig. 1:**
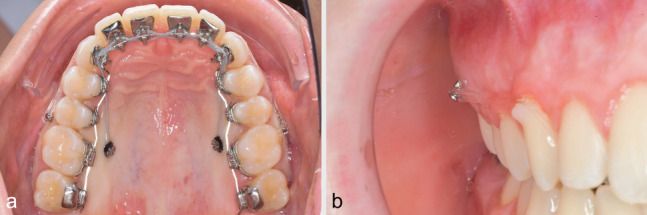
MTAD with 4 interradicular MSs (**a**). The palatal screws are placed close to the palatal roots of the first molars. The buccal MSs are inserted with a visible cranial orientation (**b**)

Concerning the orthodontic biomechanics for MTAD, in contrast to the method presented by Park et al. [6], two mini-screws per side were inserted. The entire maxillary dentition was moved simultaneously in a posterior direction using two mini-screws per side to which elastic chains (Morita Energy Chain, Rocky Mountain Orthodontics, Denver, CO, USA) were attached (Fig. 1a). The traction force per screw did not exceed 150–200 cN, as an excessive tipping moment may result in loose or lost screws [7, 16]. A 0.016″ × 0.024″ stainless steel archwire (ribbon-wise) with 2 cm expansion at the first molars and a 13 or 21° palatal root-torque from upper canine to canine was used for the maxillary distalization. Considering the limited interradicular space, the buccal screws (Abso Anchor SH 1312–10, Tiger Dental, Hörbranz, Austria) were removed 3–5 months after the start of retraction, in order not to interfere with distal tooth movements. The palatal screw (Dual Top S16-G2–010 N, Tiger Dental, Hörbranz, Austria) was inserted close to the palatal molar root, i.e. 1.5 mm distal to the midline between the second premolar and the first molar (Fig. 1a). The palatal screws were inserted perpendicular to the alveolar process and the buccal screws were angulated cranially (Fig. 1b) to position the tip of the screws in an area of greater interradicular bone [17, 18]. Class II elastics were prescribed only at nighttime to support the anterior-posterior correction. The MTAD was discontinued when a sagittal overcorrection of about 1 mm was achieved. All MSs were placed by one operator (F.B.) with high clinical expertise in the field of inserting interradicular MSs in this region (more than 250 MSs inserted per year).

The measurements according to the ABO MGS were made on the plaster models before (T1) and after orthodontic treatment (T2B) as well as on the target set-up (T2A). Along with the ABO MGS assessments, measurements of alignment and rotations, marginal ridges, buccolingual inclinations, occlusal contacts, occlusal relationship, overjet, and interproximal contacts were included (Table 1). Furthermore, the overbite and the anterior-posterior relationship at the first molar were measured in millimeters using a digital caliper. As in previous studies in which final occlusal outcomes were compared to an individual set-up, no radiographs were assessed for root parallelism [11, 12, 19]. All measurements were taken by the same investigator (Y.J.) who had successfully completed the ABO calibration directed by the former ABO Director (P.F.F.). The ABO passing score was set to 25 penalty points.

**Table 1:** Description of the measurements and intrarater reliability

Measurement	Description	ICC
Alignment	Assessment of tooth alignment. Incisal edges and lingual surfaces of maxillary anterior teeth, incisal edges and labial-incisal surfaces of mandibular anterior teeth, mesiodistal central grooves of posterior maxillary teeth and buccal cusps of posterior mandibular teeth should be in line.	0.995
Marginal ridges	Assessment of vertical positioning of posterior teeth. Marginal ridges of adjacent teeth should be at the same level.	0.845
Buccolingual inclination	Assessment of buccolingual inclination of posterior teeth. Upper and lower buccal and lingual cusps should be at the same height.	0.926
Occlusal contacts	Assessment of intercuspation of opposing teeth. The functioning cusps should be contacting the occlusal surfaces of opposing teeth.	0.973
Occlusal relationship	Assessment of anterior- posterior position of posterior teeth. The occlusion should be an Angle Class I relationship.	0.999
Overjet	Assessment of anterior-posterior relationship of anterior teeth and transverse relationship of posterior teeth. Anterior teeth should be in contact and posterior functioning cusps should be in the fossae of opposing teeth.	0.987
Interproximal contacts	Assessment of spacing within the dental arch. All teeth should be in contact with one another.	0.995
Total score	Sum of the of grading scores for the above parameters. Total score should be as low as possible.	0.997
Overbite	Measurement [mm] between two antagonistic incisors comprising the greatest vertical overlap. Overbite should be 1–2 mm.	0.991
A-P	Measurement [mm] of the discrepancy of the mesiobuccal cusp of the upper first molar in relation to the buccal central groove of the lower first molar. Anterior-posterior relation should be 0 mm.	0.999

ICC <  0.5: poor reliability; 0.5 ≤ ICC <  0.75: moderate reliability; 0.75 ≤ ICC <  0.9: good reliability; ICC ≥ 0.9: excellent reliability. A-P: anterior-posterior relationship at first molar

### Statistical analysis

Intrarater reliability was evaluated using intraclass correlation coefficients (ICC). For this purpose, 10% of the sample (8 patients) were randomly selected and remeasured after at least 2 weeks by the main investigator (Y.J.). ICC estimates were calculated based on a single measurement, absolute-agreement, 2-way mixed effects model. Interpretation of the correlation coefficients followed the cut-off limits of Koo and Li 2016 [20]. Descriptive statistics were calculated for all variables. Non-parametric tests were used since the data was not normally distributed, as assessed by the Shapiro-Wilk test (*p* <  0.05). Wilcoxon signed-rank tests were used to investigate intragroup differences and Mann-Whitney U tests were used to evaluate intergroup differences. The significance level was set to α = 5%, and a *p*-value < 0.05 was considered significant. To assess any potential dependencies of the outcome at T2B and the starting conditions at T1 the linear correlation (r) as well as the Coefficient of determination (r^2^) were derived. The datasets were analyzed using IBM SPSS Statistics 29 (IBM Corp., Armonk, NY, USA).

## Results

Forty Class II patients met the inclusion criteria. After matching the control group for age and gender, 80 patients were included in the analysis: 40 patients in Group 1 with Class I malocclusion (f/m 33/7; mean age 30.5 ± 10.0 years) and 40 patients in Group 2 with Class II malocclusion (f/m 33/7; mean age 32.6 ± 12.0 years). The baseline characteristics are summarized in Table 2.

**Table 2:** Baseline characteristics

Characteristic	Group 1 (Class I)	Group 2 (Class II)
Age (years) Mean ± SD	30.51 ± 10.03	32.62 ± 11.95
Total treatment time (years) Mean ± SD	1.29 ± 0.52	2.35 ± 0.65
Duration MTAD (months) Mean ± SD		10.20 ± 5.10
Gender n (%)		
Female	33 (82.5%)	33 (82.5%)
Male	7 (17.5%)	7 (17.5%)
Localization Class II n (%)		
Bilateral		32 (80.0%)
Unilateral		8 (20.0%)

*MTAD* Maxillary total arch distalization

**Fig. 2:**
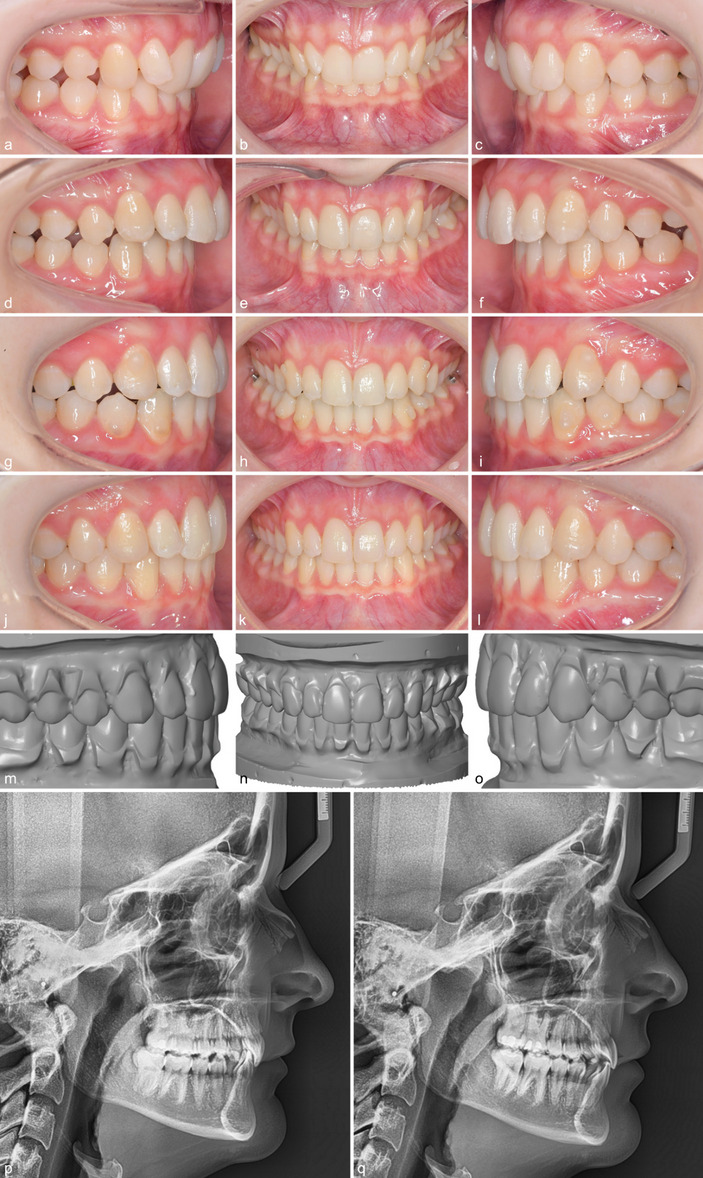
19-year-old female patient with a Class II division 2 malocclusion, deep overbite and an initial ABO MGS score of 64 (**a**-**c**). After bonding of the CCLA the Class II relationship has worsened on both sides (**d**-**f**). At the end of MTAD an overcorrection could be achieved (**g**-**i**). At the end of fixed appliance therapy, a final ABO MGS score of 14 could be achieved. The result and the individual treatment plan (target set-up with a score of 11) look very similar (**j**-**o**). The lateral headfilms before and after show a clockwise rotation of the occlusal plane with a maxillary posterior intrusion (**p**, **q**). Good levelling of the mandibular curve of Spee and acceptable torque control could be achieved with the CCLAs

**Fig. 3:**
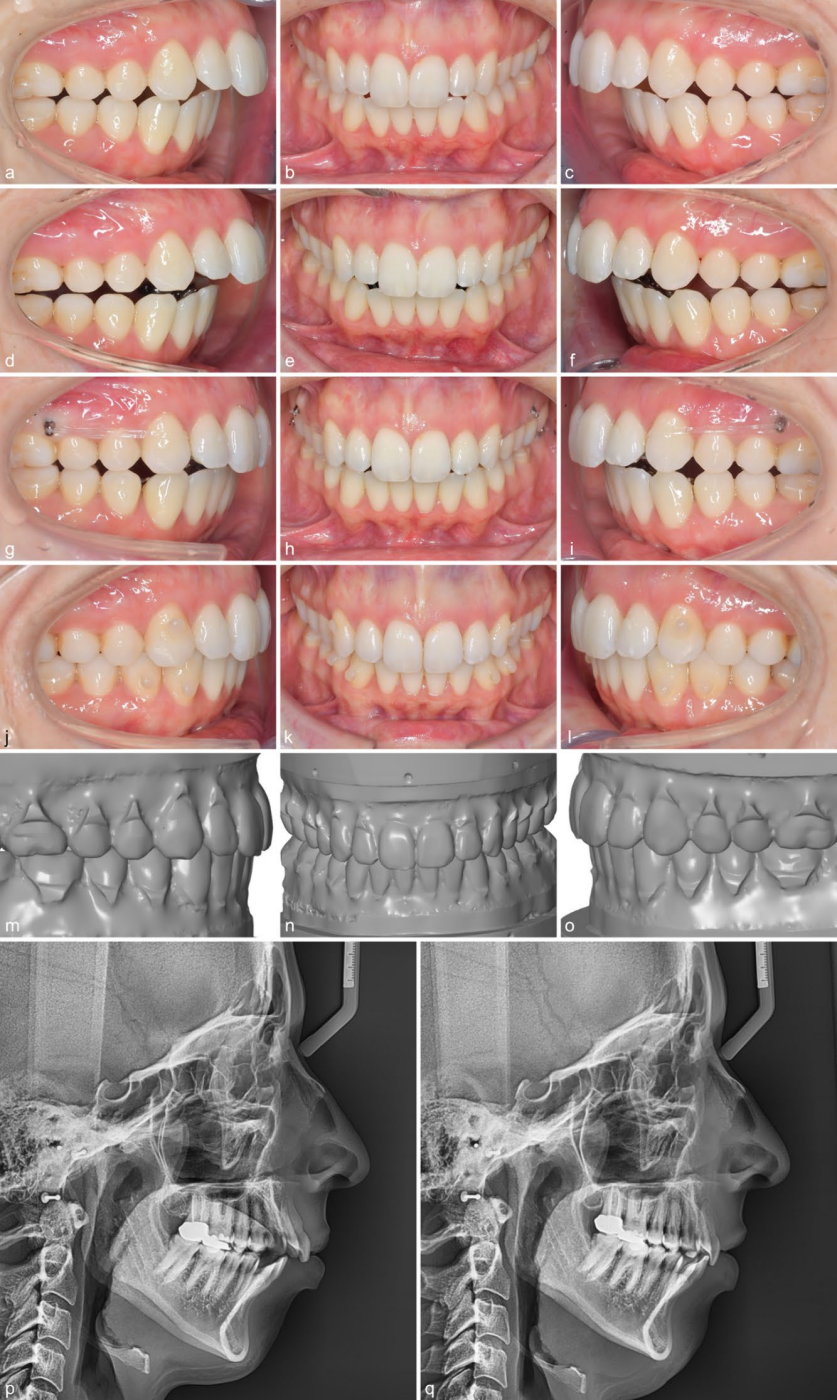
35-year-old female patient with a Class II division 1 malocclusion, an anterior open bite and an initial ABO MGS score of 57 (**a-c**). After bonding of the CCLA the Class II relationship has worsened on both sides (**d-f**). When using MTAD, further proclination of the lower incisors during Class II correction can be avoided (**g-i**). At the end of fixed appliance therapy, a final ABO MGS score of 10 could be achieved. Directly after debonding, upper and lower fixed 4–4 retainers were bonded. The patient had to wear up and down elastics in the canine region at night for 6 months in order to retain the vertical correction. The result and the individual treatment plan (target set-up with a score of 6) look very similar (**j-o**). The lateral headfilms before and after also show a clockwise rotation of the occlusal plane with a maxillary posterior intrusion (**p**, **q**). Due to this intrusion, a slight counter-clockwise rotation of the mandible can be noted. Further proclination of the lower incisors could be prevented. For better long-term stability and further improvement of the profile a genioplasty was recommended

**Fig. 4:**
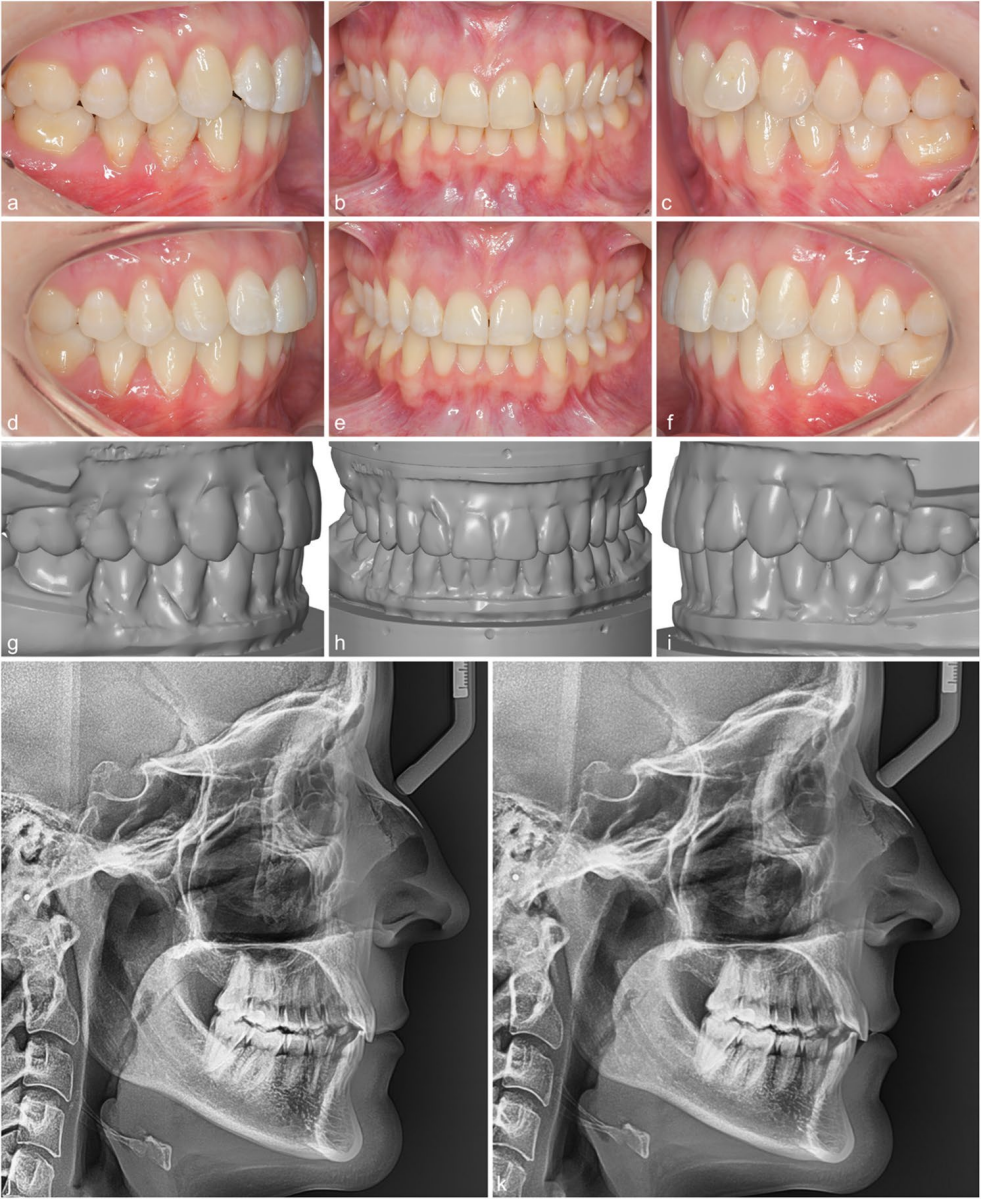
27-year-old female patient with a Class I malocclusion, upper and lower frontal crowding and an initial ABO MGS score of 37 (**a-c**). At the end of fixed appliance therapy, a final ABO MGS score of 19 could be achieved (**d-f**). The result and the individual treatment plan (target set-up with a score of 16) look very similar (**g**-**i**). The lateral headfilms before and after also show an improved inter-incisor angle (**j**, **k**)

Intra-rater reliability was excellent for all variables except for marginal ridges, which showed good reliability (Table 1). The total treatment time was on average 1.29 ± 0.52 years in the Class I group versus 2.35 ± 0.65 years in the Class II group (Table 2). 144 interradicular mini-screws were placed for bilateral distalization (32 patients) and unilateral distalization (8 patients). The mean time of MTAD with the interradicular MSs was 10.20 ± 5.10 months. Descriptive statistics for the ABO MGS and the metric measurements at T1, T2A, and T2B and comparisons between time points are shown in Tables 3, 4 and 5 for patients with Class I and Class II malocclusions, respectively (Figs. 2, 3 and 4).

**Table 3:** Class I descriptives and Wilcoxon signed-rank test statistics

																Wilcoxon signed-rank test	
	T1					T2A					T2B					T1-T2B	T2A-T2B
Variables	Mean	SD	95% CI	Min	Max	Mean	SD	95% CI	Min	Max	Mean	SD	95% CI	Min	Max	Sig	Sig
Total score	39.43	10.88	35.95–42.90	20	61	11.58	4.56	10.12–13.03	4	22	17.73	5.71	15.90–19.55	8	31	< 0.001	< 0.001
AR	21.80	6.24	19.80–23.80	11	39	2.05	2.32	1.31–2.79	0	12	4.65	2.35	3.90–5.40	1	12	< 0.001	< 0.001
MR	4.00	2.33	3.25–4.75	0	9	2.73	1.72	2.17–3.28	0	6	3.33	1.93	2.71–3.94	0	8	0.044	0.060
BI	4.22	2.61	3.39–5.06	0	11	2.18	2.40	1.41–2.94	0	10	4.23	2.49	3.43–5.02	0	11	0.903	< 0.001
OJ	4.65	3.72	3.46–5.84	0	18	0.73	1.13	0.36–1.09	0	4	1.85	1.53	1.36–2.34	0	8	< 0.001	< 0.001
OC	1.30	2.46	0.51–2.09	0	13	1.23	1.93	0.61–1.84	0	8	1.20	1.98	0.57–1.83	0	9	0.661	0.759
OR	2.95	2.75	2.07–3.83	0	10	2.73	2.14	2.04–3.41	0	8	2.40	2.07	1.74–3.06	0	8	0.109	0.331
IC	0.53	1.34	0.10–0.95	0	6	0.00	0.00	0.00–0.00	0	0	0.08	0.35	−0.04 - 0.19	0	2	0.051	0.180
OvB	3.06	1.71	2.52–3.61	0.00	8.20	2.17	0.50	2.01–2.33	1.00	3.10	2.17	0.55	2.00–2.35	1.00	3.50	0.002	0.708
A-P	−0.05	0.75	−0.29 - 0.19	−2.50	1.50	0.05	0.63	−0.16 - 0.25	−1.50	2.00	−0.13	0.60	−0.32 - 0.06	−2.00	1.10	0.656	0.042

*SD* standard deviation, *95% CI* 95% confidence interval for mean, *Sig* significance (*p*-value), *AR* alignment, *MR* marginal ridges, *BI* buccolingual inclination, *OJ* overjet, *OC* occlusal contacts, *OR* occlusal relationship, *IC* interproximal contacts, *OvB* Overbite (mm), *A-P* anterior-posterior relationship at first molar (mm)

**Table 4:** Class II descriptives and Wilcoxon signed-rank test statistics

																Wilcoxon signed-rank test	
	T1					T2A					T2B					T1-T2B	T2A-T2B
Variables	Mean	SD	95% CI	Min	Max	Mean	SD	95% CI	Min	Max	Mean	SD	95% CI	Min	Max	Sig	Sig
Total score	55.85	9.65	52.76–58.94	41	82	7.10	3.33	6.04–8.16	2	16	17.05	4.69	15.55–18.55	6	29	< 0.001	< 0.001
AR	21.07	5.45	19.33–22.82	9	35	0.85	0.92	0.56–1.14	0	4	3.33	2.16	2.63–4.02	0	9	< 0.001	< 0.001
MR	4.82	1.81	4.25–5.40	2	9	2.75	1.30	2.34–3.16	0	6	4.03	1.67	3.49–4.56	1	7	0.065	< 0.001
BI	3.47	2.43	2.70–4.25	0	9	0.45	0.75	0.21–0.69	0	3	2.85	1.92	2.24–3.46	0	8	0.128	< 0.001
OJ	8.55	4.41	7.14–9.96	2	20	0.37	1.19	−0.01 - 0.76	0	7	2.23	1.78	1.66–2.79	0	6	< 0.001	< 0.001
OC	1.47	2.57	0.65–2.30	0	11	0.30	0.65	0.09–0.51	0	2	1.48	1.96	0.85–2.10	0	7	0.784	< 0.001
OR	15.63	4.11	14.31–16.94	8	20	2.38	1.72	1.82–2.93	0	6	3.23	2.81	2.32–4.13	0	11	< 0.001	0.102
IC	0.70	1.47	0.23–1.17	0	5	0.00	0.00	0.00–0.00	0	0	0.08	0.27	−0.01 - 0.16	0	1	0.012	0.083
OvB	4.25	2.17	3.56–4.95	0.90	11.80	2.56	0.61	2.36–2.76	1.00	3.60	2.35	0.72	2.12–2.58	0.70	3.70	< 0.001	0.056
A-P	4.63	1.09	4.28–4.98	3.50	8.60	0.10	0.41	−0.03 - 0.23	0.00	2.00	0.16	0.47	0.01–0.31	0.00	2.00	< 0.001	0.077

*SD* standard deviation, *95% CI* 95% confidence interval for mean, *Sig* significance (*p*-value), *AR* alignment, *MR* marginal ridges, *BI* buccolingual inclination, *OJ* overjet, *OC* occlusal contacts, *OR* occlusal relationship, *IC* interproximal contacts, *OvB* Overbite (mm), *A-P* anterior-posterior relationship at first molar (mm)

**Table 5:** Intergroup Mann-Whitney-U test statistics

	T1	T2A	T2B
Variables	Sig	Sig	Sig
Total score	< 0.001	< 0.001	0.552
AR	0.606	< 0.001	0.013
MR	0.114	0.801	0.083
BI	0.167	< 0.001	0.007
OJ	< 0.001	0.040	0.425
OC	0.739	0.006	0.342
OR	< 0.001	0.565	0.255
IC	0.706	1.000	0.671
OvB	0.007	< 0.001	0.174
A-P	< 0.001	0.588	0.053

*Sig* significance (p-value), *AR* alignment, *MR* marginal ridges, *BI* buccolingual inclination, *OJ* overjet; *OC* occlusal contacts, *OR* occlusal relationship, *IC* interproximal contacts, *OvB* Overbite, *A-P* anterior-posterior relationship at first molar

### ABO score

**Fig. 5:**
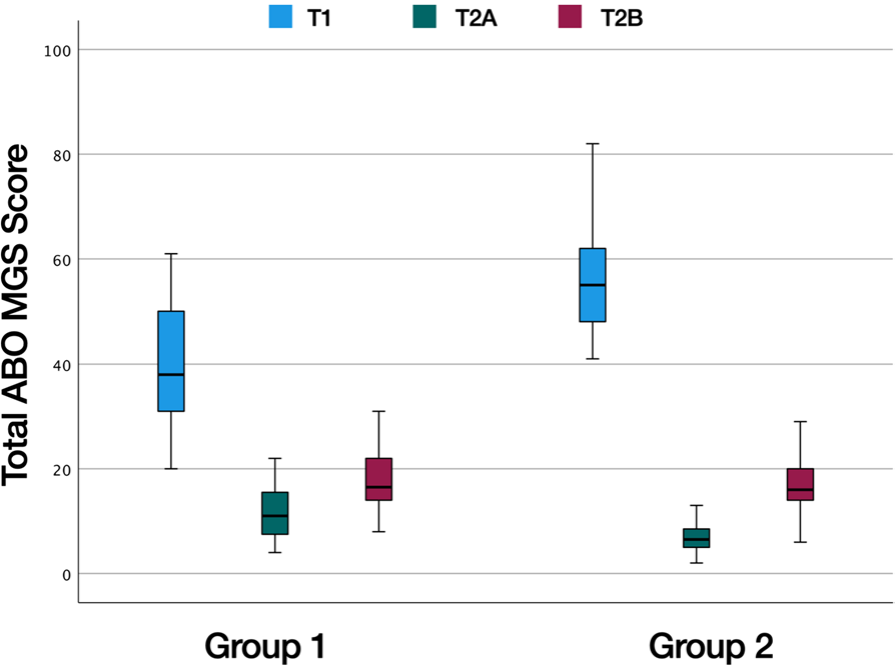
Total ABO MGS scores before treatment (T1), on the target set-up (T2A) and after orthodontic treatment (T2B) in Group 1 and 2

Assuming a passing threshold score of 25 penalty points or lower, all individual target set-ups (T2A) would meet ABO standards in both groups. Posttreatment (T2B), 38 out of 40 Class I cases (95%) and 38 out of 40 Class II cases (95%) would pass. The total ABO MGS scores of the cases that would not have passed were 31/30 in Group 1 and 29/27 in Group 2. In both groups, all OGS categories improved from pretreatment to posttreatment. There were substantial improvements in total scores in both groups and at the end of fixed lingual appliance treatment, the mean ABO MGS score was reduced from 39.4 to 17.7 in Group 1 and from 55.9 to 17.1 in Group 2 (Fig. 5). However, despite major improvements in final mean ABO MGS scores in both groups, a statistically significant difference remained between the planned (T2A) and the achieved (T2B) total ABO MGS scores. Looking at the different criteria in Group 1, there was no statistically significant difference between predicted and achieved scores for marginal ridges, occlusal contacts, occlusal relationship, interproximal contacts and overbite. In Group 2, there was no statistically significant difference between predicted and achieved scores for occlusal relationship, interproximal contacts, overbite and anterior-posterior linear measurements at the first molars.

### Metric measurements

Looking at the metric measurements, there was no statistically significant difference between predicted and achieved AP correction at the first molars in the Class II group (4.53 mm/4.47 mm), as 99% of the planned AP correction was achieved (Table 6). The mean overbite improved by 0.89 mm in Group 1, which was 100% of the planned correction. In Group 2, the observed overbite correction was 1.91 ± 2.03 mm, while the expected overbite correction was 1.69 ± 2.14 mm, indicating a degree of overcorrection (132%) for Class II patients (Table 6). There was no statistically significant difference in the overbite correction achieved between the two groups (Table 6).

**Table 6:** Anterior-posterior relationship and overbite millimetric measurements

	Class I					Class II					Mann-Whitney- U test
Variables	Mean	SD	95% CI	Min	Max	Mean	SD	95% CI	Min	Max	Sig
A-P											
Predicted (T1-T2A)	−0.09	0.58	−0.28 - 0.09	−2.00	1.50	4.53	1.08	4.19–4.88	2.00	8.60	< 0.001
Achieved (T1-T2B)	0.08	0.65	−0.12 - 0.29	−1.20	3.50	4.47	1.09	4.13–4.82	2.10	8.60	< 0.001
Achieved (T1-T2B)/(T1-T2A) %	72.78					98.81					0.031
OvB											
Predicted (T1-T2A)	0.89	1.63	0.37–1.41	−2.00	5.40	1.69	2.14	1.00–2.38	−1.40	9.40	0.099
Achieved (T1-T2B)	0.89	1.54	0.40–1.38	−2.00	4.70	1.91	2.03	1.26–2.55	−1.70	9.40	0.017
Achieved (T1-T2B)/(T1-T2A) %	111.69					132.13					0.640

*SD* standard deviation, *95% CI* 95% confidence interval for mean, *Sig* significance (*p*-value), *A-P* anterior-posterior relationship at first molar (mm), *OvB* Overbite (mm)

### Intergroup comparison

The intergroup comparison of ABO MGS measurements showed higher total scores in Group 2 at T1, mainly due to higher scores for the occlusal relationship (Tables 3 and 4). In the individual target set-up (T2A), slightly lower total scores were found in Group 2. Looking at the different components, the treatment results in the Class II group were similar to the results in Group 1, except for the alignment and buccolingual inclination scores, which were slightly better in the Class II group.

### Correlation between initial and final molar relationship

**Fig. 6:**
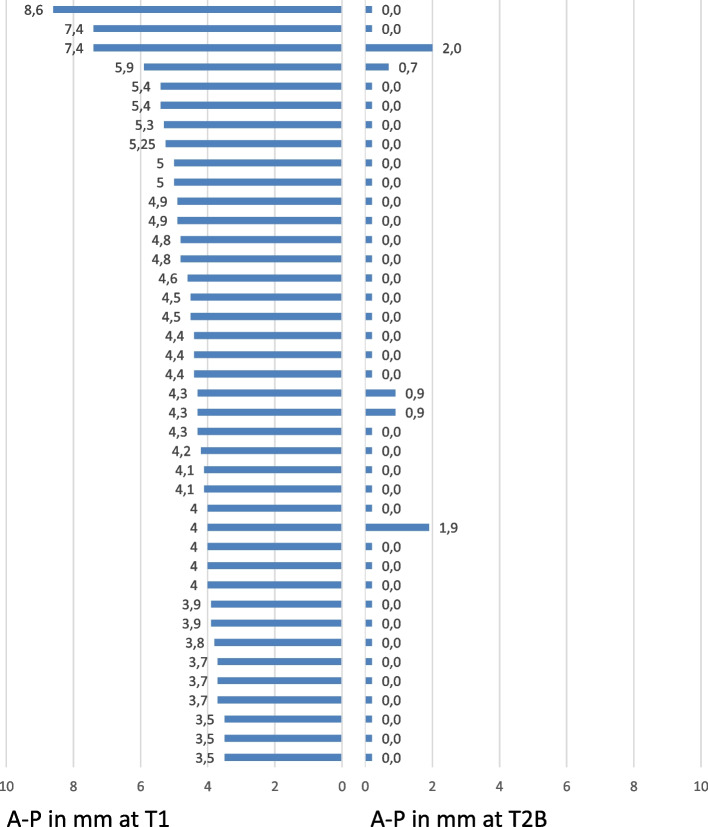
Anterior-posterior relationship at the first molar (A-P) in Group 2 in millimeters before (T1) and after orthodontic treatment (T2B). The diagram shows no correlation between the initial and final anterior-posterior relationship

Figure 6 shows an overview of the initial severity of the distal relationship at the first molar (T1) and the final correction achieved (T2B) in the Class II group. No correlation was found between the initial severity of the distal occlusion and the quality of the final anterior-posterior occlusal relationship (correlation r = 0.23, r^2^ = 0.0529).

### Mini-screw survival rate

A total of 144 interradicular mini-screws were placed for MTAD. Three of them were lost before schedule, indicating a survival rate of 97%. None of the lost mini-screws had to be replaced as the second one in the relevant quadrant was serviceable until the end of simultaneous total arch distalization. In one patient, a palatal screw had to be relocated one segment further distal as it was an obstacle for complete bite correction.

## Discussion

This study is the first to evaluate the quality of the occlusal outcome with total arch distalization using ABO MGS scores. Previous studies of mini-screw-supported maxillary distalization have primarily used cephalograms or digital 3D casts to evaluate the amount of distalization in the maxillary first molar region, along with any first molar tipping [21, 22]. Numerous papers on mini-screw-supported maxillary distalization include in their analysis the first treatment stage only, meaning the distalization of the upper molars [3, 4, 23–33]. The outcome of the subsequent retraction of the anterior segment that such an approach requires to achieve a Class I canine relationship, was not included in many cases. Beyling et al. were the first to describe the MTAD results in relation to the lower jaw based on the canine relationship and overjet corrections and to compare them to the intended outcome as defined by the individual target set-up [10]. In the present study, the dentoalveolar correction of the Class II from the upper jaw was also achieved by using a CCLA combined with interradicular MSs according to the method described by Beyling et al. [10]. For more in-depth assessment of the treatment outcome, the Class II patients were compared to a Class I control group matched for age and gender. The null hypothesis was rejected: There was no significant difference in the quality of the treatment outcome between a group of patients with a Class II malocclusion treated with MSs for maxillary total arch distalization and a Class I group.

The average sagittal correction achieved in the area of the first molars amounted to 4.5 mm, representing an outcome of 99% of what had been intended (T2A). Earlier studies have shown that when lingual appliances are used in patients exhibiting Class II malocclusion, the clockwise rotation of the lower jaw immediately after indirect bonding will result in a sagittal relationship that is worse by 1 mm on average (Figs. 2 and 3) [10, 34, 35]. Considering the intended overcorrection of 1 mm, an average total correction of more than 6 mm can be assumed at the end of the MTAD phase. In this regard, the success of the sagittal correction has been demonstrated not to depend on the initial severity of the class II relationship (Fig. 5). This emphasises the effectiveness and efficiency of the selected method compared to alternative MTAD approaches [23, 24]. One essential precondition for successful sagittal correction is the comprehensive levelling of the lower curve of Spee and the overbite correction associated. There is ample evidence that CCLAs are very effective for this purpose [10, 34–36]. No statistically significant difference was found at the end of treatment (T2B) between both groups, while the bite raising (T2A) in the Class II group even exceeded what had been intended by 0.2 mm on average (Table 6). The mean correction of the overbite in Group 1 was 0.89 mm. This is because both open and deep bites were included. Nevertheless, the overbite could be fully corrected as planned in the set-up. The fact that there was a mathematical overcorrection of the overbite 113% in Group 1 is somewhat diminished clinically, as open and deep bites were included, and the range was large.

With a comparable approach and method, Patterson et al. and Leavitt described the outcome of aligner treatment in adult patients with Class II malocclusion and compared the post-treatment results to a matched group of Class I patients [11, 12]. As opposed to the outcomes in this study, the adequate outcome represented by the Class I patients could not be achieved in the preselected Class II patients despite good compliance in using intermaxillary elastics, neither with the first set of aligners nor after more than 3.5 refinements on average [11, 12]. After the first set of aligners, not only unsuccessful sagittal correction was found, but both groups also had significantly worse scores for occlusal contacts (> 10 penalty points) [11]. The evaluation of the same component in this study, on the contrary, yielded a particularly good result with less than 1.5 penalty points in both groups at T2B.

Looking at the MTAD per se, the most astounding aspect is the simplicity of the distalization mechanics placed in this study and its convincing efficiency. Interradicular insertion allows direct use of the anchorage and eliminates the need for a supra-construction. The screw loss rate in this study of less than 3% is also a consequence of the operator learning curve already described by Berens et al. for the insertion of the interradicular MSs in the relevant areas [37]. These last two points add up to a practical and pragmatic approach.

### Strengths and limitations

The retrospective nature of this study is the result of its innovative approach to MTAD. Strict inclusion and exclusion criteria helped to minimize the risk of bias. No patient was excluded from this retrospective analysis for any reason other than the defined exclusion criteria, i.e., no exclusion due to missed appointments, lack of compliance, or missing records, as is occasionally seen in sample compositions of retrospective studies.

The evaluation of the quality of the occlusal outcome after orthodontic treatment using the criteria by the American Board of Orthodontics is one of the few methods, along with the PAR index, for assessing the occlusion after orthodontic treatment as objectively as possible. Despite individual rater calibration, a subjective component in the evaluation, albeit small, cannot be ruled out. In previous studies on the quality of the occlusion after CCLA treatment, different raters, who had also been calibrated, found above-average outcomes [19, 34, 38, 39]. The results of this study confirm that the treatment standard is above average.

In both Groups a statistically significant difference in total ABO scores between T2A and T2B became obvious. As previously defined, T2A represents the patient specific ideal occlusion defined by the individual target set-up. Theoretically, an ideal appliance would correct the occlusion comparable to a mathematical “function of a limit” more and more towards the individual ideal situation, making the differences between both of them smaller and smaller over time. The high quality of the final occlusions achieved in this investigation underlines the thoughtful definition of the endpoint of active lingual fixed appliance therapy despite a statistically significant difference in the scores at T2A and T2B.

Many previous studies into MTAD assessed the outcome quality based exclusively on the amount of distalization achieved in the maxilla, not considering the posttreatment occlusion [21, 22]. They relied on the analysis of superimposed lateral headfilms and/or digital casts of the upper jaw. The question this raises, as to the occlusal quality of the treatment outcome achievable by a distalization of this kind, could be answered in this study.

The study was conducted in a single orthodontic practice in Bad Essen, Germany. The results may therefore not be fully generalizable to other orthodontic settings. In addition, all mini-screws were placed by a single experienced operator (FB), which could affect the reproducibility of results.

## Conclusions

The quality of the occlusal outcome after maxillary total arch distalization with a completely customized lingual appliance and interradicular mini-screws is high even in severe cases and the amount of the sagittal correction can be predicted well by the individual target set-up.

## Data Availability

The data underlying this study can be shared upon reasonable request to the corresponding author.

## References

[1] Umalkar SS, Jadhav VV, Paul P, Reche A (2022). Modern Anchorage Systems in Orthodontics. Cureus..

[2] Kinzinger GS, Gülden N, Yildizhan F, Diedrich PR (2009). Efficiency of a skeletonized distal jet appliance supported by miniscrew anchorage for noncompliance maxillary molar distalization. Am J Orthod Dentofacial Orthop..

[3] Nienkemper M, Wilmes B, Pauls A, Yamaguchi S, Ludwig B, Drescher D (2014). Treatment efficiency of mini-implant-borne distalization depending on age and second-molar eruption. J Orofac Orthop..

[4] Wilmes B, Drescher D (2010). Application and effectiveness of the Beneslider: a device to move molars distally. World J Orthod..

[5] Bae SM, Park HS, Kyung HM, Kwon OW, Sung JH (2002). Clinical application of micro-implant anchorage. J Clin Orthod..

[6] Park HS, Kwon TG, Sung JH (2004). Nonextraction treatment with microscrew implants. Angle Orthod..

[7] Wiechmann D, Meyer U, Büchter A (2007). Success rate of mini- and micro-implants used for orthodontic anchorage: a prospective clinical study. Clin Oral Implants Res..

[8] Graf S, Vasudavan S, Wilmes B (2018). CAD-CAM design and 3-dimensional printing of mini-implant retained orthodontic appliances. Am J Orthod Dentofacial Orthop..

[9] Park HS, Bae SM, Kyung HM, Sung JH. Simultaneous incisor retraction and distal molar movement with microimplant anchorage. World J Orthod. 2004;5(2):164–71. 15615135

[10] Beyling F, Klang E, Niehoff E, Schwestka-Polly R, Helms HJ, Wiechmann D. Class II correction by maxillary en masse distalization using a completely customized lingual appliance and a novel mini-screw anchorage concept - preliminary results. Head Face Med. 2021;17(1):23. 10.1186/s13005-021-00273-3 PMC8240392 34187487

[11] Patterson BD, Foley PF, Ueno H, Mason SA, Schneider PP, Kim KB. Class II malocclusion correction with Invisalign: Is it possible? Am J Orthod Dentofacial Orthop. 2021;159(1):e41–8. 10.1016/j.ajodo.2020.08.016 33223374

[12] Leavitt TD. An evaluation of Invisalign treatment comparing Class I and Class II malocclusion, using the American Board of Orthodontics objective grading system. [Master's thesis]. Saint Louis: Saint Louis University; 2019.

[13] Pauls AH. Therapeutic accuracy of individualized brackets in lingual orthodontics. J Orofac Orthop. 2010;71(5):348–61. 10.1007/s00056-010-1027-3 20963544

[14] Grauer D, Proffit WR. Accuracy in tooth positioning with a fully customized lingual orthodontic appliance. Am J Orthod Dentofacial Orthop. 2011;140(3):433–43. 10.1016/j.ajodo.2011.01.020 21889089

[15] Pauls A, Nienkemper M, Schwestka-Polly R, Wiechmann D. Therapeutic accuracy of the completely customized lingual appliance WIN : A retrospective cohort study. J Orofac Orthop. 2017;78(1):52–61. 10.1007/s00056-016-0058-9 PMC5247553 27858111

[16] Büchter A, Wiechmann D, Gaertner C, Hendrik M, Vogeler M, Wiesmann HP, Piffko J, Meyer U. Load-related bone modelling at the interface of orthodontic micro-implants. Clin Oral Implants Res. 2006;17(6):714–22. 10.1111/j.1600-0501.2006.01233.x 17092232

[17] Park HS, Kim JY, Kwon TG. Treatment of a Class II deepbite with microimplant anchorage. Am J Orthod Dentofacial Orthop. 2011;139(3):397–406. 10.1016/j.ajodo.2009.02.034 21392696

[18] Yoon JH, Cha JY, Choi YJ, Park WS, Han SS, Lee KJ. Simulation of miniscrew-root distance available for molar distalization depending on the miniscrew insertion angle and vertical facial type. PLoS One. 2020;15(9):e0239759. 10.1371/journal.pone.0239759 PMC7514046 32970759

[19] AlQatami FM, Alouini O, Knösel M, Helms HJ, Schwestka-Polly R. Objective treatment outcome assessment of a completely customized lingual appliance: A retrospective study. Int Orthod. 2021;19(3):445–52. 10.1016/j.ortho.2021.06.004 34305012

[20] Koo TK, Li MY. A Guideline of Selecting and Reporting Intraclass Correlation Coefficients for Reliability Research. J Chiropr Med. 2016;15(2):155–63. 10.1016/j.jcm.2016.02.012 PMC4913118 27330520

[21] Ceratti C, Serafin M, Del Fabbro M, Caprioglio A. Effectiveness of miniscrew-supported maxillary molar distalization according to temporary anchorage device features and appliance design: systematic review and meta-analysis. Angle Orthod. 2024;94(1):107–21. 10.2319/052223-364.1 PMC10928936 37870251

[22] Raghis TR, Alsulaiman TMA, Mahmoud G, Youssef M. Efficiency of maxillary total arch distalization using temporary anchorage devices (TADs) for treatment of Class II-malocclusions: A systematic review and meta-analysis. Int Orthod. 2022;20(3):100666. 10.1016/j.ortho.2022.100666 35871982

[23] Cozzani M, Fontana M, Maino G, Maino G, Palpacelli L, Caprioglio A. Comparison between direct vs indirect anchorage in two miniscrew-supported distalizing devices. Angle Orthod. 2016;86(3):399–406. 10.2319/040715-231.1 PMC8601749 26222412

[24] Duran GS, Görgülü S, Dindaroğlu F. Three-dimensional analysis of tooth movements after palatal miniscrew-supported molar distalization. Am J Orthod Dentofacial Orthop. 2016;150(1):188–97. 10.1016/j.ajodo.2015.12.024 27364220

[25] Escobar SA, Tellez PA, Moncada CA, Villegas CA, Latorre CM, Oberti G. Distalization of maxillary molars with the bone-supported pendulum: a clinical study. Am J Orthod Dentofacial Orthop. 2007;131(4):545–9. 10.1016/j.ajodo.2006.08.012 17418723

[26] Gelgör IE, Büyükyilmaz T, Karaman AI, Dolanmaz D, Kalayci A. Intraosseous screw-supported upper molar distalization. Angle Orthod. 2004;74(6):838–50. 10.1043/0003-3219(2004)074<0838:ISUMD>2.0.CO;2 15673149

[27] Gelgör IE, Karaman AI, Buyükyilmaz T. Comparison of 2 distalization systems supported by intraosseous screws. Am J Orthod Dentofacial Orthop. 2007;131(2):161.e1–8. 10.1016/j.ajodo.2006.03.027 17276855

[28] Kinzinger GS, Gülden N, Yildizhan F, Diedrich PR. Efficiency of a skeletonized distal jet appliance supported by miniscrew anchorage for noncompliance maxillary molar distalization. Am J Orthod Dentofacial Orthop. 2009;136(4):578–86. 10.1016/j.ajodo.2007.10.049 19815162

[29] Kuroda S, Hichijo N, Sato M, Mino A, Tamamura N, Iwata M, Tanaka E. Long-term stability of maxillary group distalization with interradicular miniscrews in a patient with a Class II Division 2 malocclusion. Am J Orthod Dentofacial Orthop. 2016;149(6):912–22. 10.1016/j.ajodo.2015.07.045 27242002

[30] Kuroda S, Yamada K, Deguchi T, Kyung HM, Takano-Yamamoto T. Class II malocclusion treated with miniscrew anchorage: comparison with traditional orthodontic mechanics outcomes. Am J Orthod Dentofacial Orthop. 2009;135(3):302–9. 10.1016/j.ajodo.2007.03.038 19268827

[31] Mariani L, Maino G, Caprioglio A. Skeletal versus conventional intraoral anchorage for the treatment of class II malocclusion: dentoalveolar and skeletal effects. Prog Orthod. 2014;15(1):43. 10.1186/s40510-014-0043-z PMC4138549 25138818

[32] Polat-Ozsoy O, Kircelli BH, Arman-Ozçirpici A, Pektaş ZO, Uçkan S. Pendulum appliances with 2 anchorage designs: conventional anchorage vs bone anchorage. Am J Orthod Dentofacial Orthop. 2008;133(3):339.e9–339.e17. 10.1016/j.ajodo.2007.10.002 18331928

[33] Sar C, Kaya B, Ozsoy O, Özcirpici AA. Comparison of two implant-supported molar distalization systems. Angle Orthod. 2013;83(3):460–7. 10.2319/080512-630.1 PMC8763062 23106546

[34] Vu J, Pancherz H, Schwestka-Polly R, Wiechmann D. Correction of Class II, Division 2 malocclusions using a completely customized lingual appliance and the Herbst device. J Orofac Orthop. 2012;73(3):225–35. 10.1007/s00056-012-0077-0 22576865

[35] Klang E, Beyling F, Knösel M, Wiechmann D. Quality of occlusal outcome following space closure in cases of lower second premolar aplasia using lingual orthodontic molar mesialization without maxillary counterbalancing extraction. Head Face Med. 2018;14(1):17. 10.1186/s13005-018-0176-2 PMC6154920 30249268

[36] Alouini O, Knösel M, Blanck-Lubarsch M, Helms HJ, Wiechmann D. Controlling incisor torque with completely customized lingual appliances. J Orofac Orthop. 2020;81(5):328–39. 10.1007/s00056-020-00231-9 PMC7442759 32472341

[37] Berens A, Wiechmann D, Dempf R. Mini- and micro-screws for temporary skeletal anchorage in orthodontic therapy. J Orofac Orthop. 2006;67(6):450–8. 10.1007/s00056-006-0601-1 17124564

[38] Mujagic M, Pandis N, Fleming PS, Katsaros C. The Herbst appliance combined with a completely customized lingual appliance: A retrospective cohort study of clinical outcomes using the American Board of Orthodontics Objective Grading System. Int Orthod. 2020;18(4):732–8. 10.1016/j.ortho.2020.07.002 32839142

[39] Graf I, Bock NC, Bartzela T, Röper V, Schumann U, Reck K, Christ H, Höfer K, Fritz U, Wiechmann D, Jost-Brinkmann PG, Wolf M, Ruf S, Braumann B. Quality of orthodontic care-A multicenter cohort study in Germany : Part 1: Evaluation of effectiveness of orthodontic treatments and predictive factors. J Orofac Orthop. 2022;83(5):291–306. 10.1007/s00056-021-00304-3 PMC9395451 34142175

